# Bioprospecting Microbial Consortia for Tebuthiuron Degradation in Agricultural Soils: An Alternative Bayesian‐Driven Colorimetric Protocol

**DOI:** 10.1111/1758-2229.70109

**Published:** 2025-05-26

**Authors:** Letícia Barbosa Jorososki, Bruno Rafael de Almeida Moreira, Victor Hugo Cruz, Yanca Araujo Frias, Paulo Renato Matos Lopes

**Affiliations:** ^1^ Department of Plant Production College of Agricultural and Technological Sciences, São Paulo State University (UNESP) Dracena São Paulo Brazil; ^2^ Centre for Crop Science, Queensland Alliance of Agriculture and Food Innovation (QAAFI) The University of Queensland (UQ) Brisbane Queensland Australia

**Keywords:** agricultural pesticide, bioaugmentation, environmental pollution, microbial bioremediation, persistent herbicide, sustainable sugarcane agroecosystems

## Abstract

Herbicides impact, particularly tebuthiuron (TBT), on agroecosystems and surrounding environments had been documented in scientific literature. This study investigated the hypothesis that areas exposed to TBT, a prevalent herbicide in Brazil's sugarcane crops, might harbour microbial consortia capable of degrading this compound, assessed through a Bayesian‐based colorimetric method. Soil samples from plant cane (pC), characterised by lower organic matter on the surface, and first‐cut ratoon cane (rC) fields, with higher organic matter due to crop residues deposition, were collected for analysis. Colorimetric assays with DCPIP (2,6‐Dichlorophenolindophenol) were performed with microorganisms isolated from these fields to evaluate their TBT‐degradation capacity. In an ELISA microplate, absorbance was measured at 600 nm as DCPIP is a redox indicator. Results confirmed the degradation potential of soil microbial consortia, particularly from rC samples, as evidenced by reduced absorbance relative to the control. The data deviated from the expected sigmoidal pattern, necessitating an alternative data interpretation method. A Bayesian factor estimation approach for kernel density curves of the logarithmic response ratio proved effective for handling non‐sigmoidal spectrophotometric data. The findings offered valuable insights into TBT‐degrading microorganisms and introduced an alternative analytical tool for interpreting variable data, potentially aiding in the development of bio‐based remediation strategies.

## Graphical abstract

Microbial consortia from sugarcane soils, particularly ratoon cane environments, showed tebuthiuron‐degrading potential. Bayesian factor estimation of kernel density curves based on logarithmic response ratios enabled interpretation of non‐sigmoidal spectrophotometric data, revealing native microbiota's bioremediation capacity and offering a novel analytical approach for developing bio‐based remediation strategies.

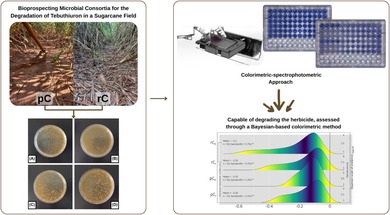

**Abbreviations**
CFU: Colony‐forming units
DCPIP: 2,6‐Dichlorophenolindophenol
KDE: Kernel density estimation
LeRR: Natural logarithmic response ratio
pC: Plant cane
PSII: Photosystem II
rC: First‐cut ratoon cane
TBT: Tebuthiuron

## 1 Introduction

The proliferation of weeds in intensive agricultural systems is typically a result of environmental alterations, predominantly instigated by human‐led crop cultivation. Native flora serves as a natural countermeasure, rectifying concerns such as mineral imbalances and soil compaction (Primavesi 2017). These disruptions can notably diminish productivity, with potential losses reaching up to 85%. The management of these elements is vital for cost efficiency and the preservation of economically significant plants (Silva et al. 2018).

Herbicides, widely recognised for their role in chemical weed control, interact with soil, plants, water and air, establishing a multifaceted dynamic (Albuquerque et al. 2016). This leads to a variety of environmental transformations and potential contamination risks (Correia 2018). Pesticides eventually settle in the soil matrix, where they can be adsorbed, become bioavailable, or undergo microbial degradation (Mendes, Mielke, et al. 2021), a process integral to managing their environmental footprint.

Tebuthiuron, a PSII targeting herbicide, interrupts electron transport, inducing oxidative stress and leading to plant mortality (Pires et al. 2005; Silva Junior et al. 2018). Chemically 1‐(5‐tert‐butyl‐1,3,4‐thiadiazol‐2‐yl)‐1,3‐dimethylurea has the molecular formula C_9_H_16_N_4_OS (Brazil 2020), and it is characterised by a half‐life (t_1_/_2_) ranging from 360 to 450 days, water solubility of 2500 mg L^−1^ at 25°C, an octanol–water partition coefficient (log Kow) of 1.79, classifying it as lipophilic, a density of 1.25 g mL^−1^, a vapour pressure of 0.27 mPa (25°C), and a non‐ionizable dissociation constant (pKa), which enhances its mobility within the soil profile. Additionally, its sorption coefficient for organic carbon content (Koc) is 80 mg L^−1^ (Mancuso et al. 2011; Rodrigues and Almeida 2011).

Moreover, tebuthiuron exhibits high sorption to soil particles with higher organic matter content (Teixeira et al. 2018). Therefore, variations in herbicide behaviour in soil due to this attribute may influence the risk of environmental contamination (Mendes, Wei, et al. 2021). Thus, persistent TBT can result in bioaccumulation and intoxication across trophic levels (Pires et al. 2005; Kapsi et al. 2019; Almeida 2020). Reports of elevated TBT residue levels near sugarcane plantations in Brazil and China (Qian et al. 2017) underscore potential risks and the necessity for additional research on soil microbial communities (Sagarkar et al. 2013; Wan et al. 2014; Zhang et al. 2014).

The escalating impact of anthropogenic activities on agroecosystems necessitates remediation strategies, and bioremediation presents an ecologically sound solution for treating contaminated areas (Mallman et al. 2019). Techniques such as biostimulation and bioaugmentation can accelerate the natural attenuation processes of microorganisms, thereby reducing the duration of biological treatments (Lima et al. 2022; Lopes et al. 2022).

This research is predicated on the hypothesis that indigenous microorganisms in agricultural soils exposed to TBT may have the metabolic capacity to degrade this herbicide, contributing to soil detoxification and restoration. This hypothesis is grounded in the assumption that microbial communities in soils with a history of TBT exposure have previously selected strains that use TBT as a carbon source in their metabolism, leading to the emergence of TBT‐degrading microbiota.

Scientific literature already confirms the presence of functional native microorganisms in TBT‐contaminated soils, particularly within sugarcane cultivation zones (Lima et al. 2022; Lopes et al. 2022). These microorganisms have demonstrated a significant capacity to adapt and flourish in such environments. Mostafa and Helling (2003) identified *Methylobacterium organophilum* , *Paenibacillus pabuli* , and *Methylobacterium radiotolerans* by partial 16S DNA sequence analysis, using the first 527 bp of the 16S rRNA gene for bacterial identification, as Tebuthiuron‐degrading microorganisms in tropical soils. Also, Lima et al. (2022) used respirometry assays to quantify their TBT degradation capacity, measuring the amount of CO_2_ produced. These data are further analysed using stoichiometric models to infer the kinetic parameters of the microbial degradation process. These models typically generate Gompertz‐like curves, which depict the progression of microbial TBT metabolism over time.

Hence, the present study introduces a colorimetric technique to expedite the identification of TBT‐degrading microorganisms and to streamline the bioprospecting process. The colorimetric technique proposed in this study offers a more cost‐effective and operationally simple alternative. It does not require advanced instrumentation and allows for rapid assessment of microbial activity. While chromatographic methods provide precise quantification of TBT and its degradation intermediates, our approach focuses on detecting microbial metabolic responses, offering a practical and scalable tool for preliminary screening in biodegradation studies. However, this protocol in unconventional domains may result in atypical data patterns, potentially challenging the results consistency. Acknowledging this possibility, it is imperative to consider the identification and utilisation of alternative analytical tools capable of effectively managing and interpreting these inconsistencies.

Therefore, this research aimed to: (i) evaluate the TBT‐degradation capabilities of indigenous microorganisms' consortia isolated from agricultural soils with a history of TBT exposure, and (ii) develop a colorimetric‐spectrophotometric approach for rapid evaluation.

## 2 Materials and Methods

### 2.1 Soil Sampling in Sugarcane Cultivation

Soil samples were obtained in March 2023 from a conventional sugarcane cultivation area with a documented history of tebuthiuron application, located in the city of Junqueirópolis, São Paulo, Brazil. Sampling involved the use of a sanitised shovel to collect soil at a depth of 15 cm from randomly designated points within a 100 m^2^ area across the fields.

Two composite samples for each prospection scenario were procured to represent varying management practices within the crop: pC, characterised by a lower deposition of organic matter on the soil surface, and rC, noted by higher organic matter input from crop residue deposition. Thus, two composite soil samples were collected in each agricultural management (pC1, pC2, rC1, and rC2). The soil sampling and soil management procedure was followed as described by Lima et al. (2022) and Filizola et al. (2006). The history of tebuthiuron application in these areas is detailed in Table 1.

**TABLE 1:** Tebuthiuron management in sugarcane fields.

Field	Planting date	Last application	Commercial product	Dose (L. ha^−1^)
Plant cane (pC)	April 2022	April 2022	Spike (Corteva Agriscience)	1.6
Ratoon cane (rC)	September 2022	November 2022	Butiron (ADAMA)	1.7

### 2.2 Isolation of Tebuthiuron‐Degrading Microorganisms

The assessment of tebuthiuron biodegradation potential by direct evaluation of the metabolic activity of microbial consortia in soil was conducted using Combine 500SC (Dow AgroSciences Industrial Ltda). Bushnell‐Haas (BH) saline medium does not have a carbon source and presented the herbicide as the substrate for the isolation of microbial consortia strains with the capacity to biodegrade this molecule. Hence, solutions contained 100 and 1.0 mg L^−1^ of tebuthiuron for the bioprospection and colorimetric analysis procedures, respectively.

Microbial consortia isolation followed the methodology adapted by the research group (Lima et al. 2022). Erlenmeyer flasks containing 90 mL of Bushnell‐Haas medium with 100 mg L^−1^ tebuthiuron (BH + TBT) and 10 g of soil were incubated at 150 rpm and 35°C for 48 h. This process was repeated three times, transferring 10 mL of culture to fresh BH + TBT medium after each incubation. Microbial growth was confirmed by culturing in Nutrient Broth at 35°C for 48 h.

All isolated colonies were included in the colorimetric experiment without a pre‐selection criterion, ensuring a comprehensive evaluation of the microbial consortia's degradation potential.

### 2.3 Microbial Activity by Colorimetry

The experimental design for analysing microbial activity was randomised (Table 2), focusing on the presence of TBT and the inoculation of isolated microorganism consortia from two different cultivation managements: plant cane (pC_1_ and pC_2_) and ratoon cane (rC_1_ and rC_2_).

**TABLE 2:** Experimental design for colorimetric analysis of microbial metabolism.

Treatment	TBT	Isolate
C. DCPIP	−	−
C. TBT	+	−
pC_1_	+	pC_1_
pC_2_	+	pC_2_
rC_1_	+	rC_1_
rC_2_	+	rC_2_

The microbial activity in the degradation of TBT was assessed using colorimetric assays with DCPIP, following the methodologies by Hanson et al. (1993) and Bidoia et al. (2010). Two control conditions were established: one with BH, DCPIP, and deionised water (C. DCPIP), and another with BH, DCPIP, and TBT (C. TBT). Both controls did not include a microbial isolates consortium.

The biodegradation effectiveness was determined by the rate of medium discoloration over time. This was measured in an ELISA microplate using a UV–Vis spectrophotometer at 600 nm. Each well of the microplate contained a mixture of 190 μL of BH medium, 10 μL of 1.0 g L^−1^ DCPIP solution, 36 μL of microbial inoculum at a concentration of 4.27 × 10^5^ CFU mL^−1^, and 54 μL of 1.0 g L^−1^ TBT. This mixture was incubated at 35°C with medium agitation, based on Skanlt RE 7.0.2 software, and the absorbance was measured at regular intervals.

### 2.4 Statistical Data Analysis

A Tukey test (significance level of 0.05) was applied to CFU data. The microbial activity in TBT degradation was quantified by calculating the LeRR, which was obtained by dividing the values from treatments involving microbial isolates by those from the negative control group (TBT only). This calculation was performed using Microsoft Excel, and the formula for LeRR (Hedges et al. 1999) is as follows:

(1) $$\text{LeRR}=\mathrm{Ln}\frac{M_{t}}{M_{c}}$$

*M*_*t*_ is the mean absorbance (or colorimetric response) of the treatment, and *M*_*c*_ is the mean absorbance of the control group (TBT only).

Subsequently, a Welch's test was used to compare these conditions. The Bayes factor was used to provide evidence for the alternative hypothesis, suggesting that at least one isolate consortium significantly contributes to TBT degradation. This was contrasted with the null hypothesis, which assumes no difference between microbial conditions. To visually represent our findings, a kernel density plot was generated, incorporating metrics from Welch and Games‐Howell tests and Bayes factor. This part of our statistical methodology was executed in R, using packages such as ‘bayesfactor’ and ‘ggplot2’. The function “t.test ( )” was also used, which performs Welch's test without assuming equal variances.

## 3 Results and Discussion

### 3.1 Bioprospection of Indigenous Soil Microbiota

Microbial growth was detected in all soil samples and showed an isolated consortium of microorganisms from both sugarcane cultivation managements. The microbes were quantified through plating, yielding an average of 4.3 × 10^6^ CFU g^−1^ of soil. The individual samples, namely pC_1_, pC_2_, rC_1_, and rC_2_, exhibited results of 4.4 × 10^6^, 4.4 × 10^6^, 4.3 × 10^6^, and 3.85 × 10^6^ CFU g^−1^ soil, respectively (Figure S1). A Tukey test revealed no significant difference among these results, which endorses the rigour of the experimental protocol by inoculating similar quantities of each microorganism in the metabolic analysis.

### 3.2 Microbial Activity in the Degradation of Tebuthiuron

**FIGURE 1:**
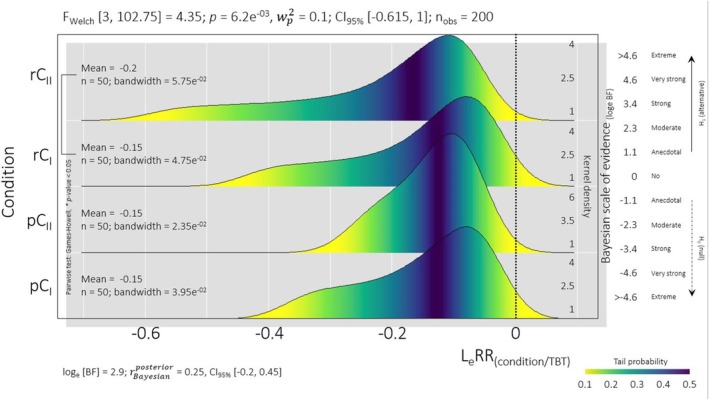
Tebuthiuron degradation by indigenous soil microbiota consortium isolated from two sugarcane managements: Plant cane (pC_1_, pC_2_) and ratoon cane (rC_1_, rC_2_). Kernel density curves representing microbial efficiency in TBT biodegradation, with negative LeRR values indicating increased metabolism. Statistical tests (Welch and Games–Howell) and Jaffrey's default Bayes factor analyse treatment differences and validate the alternative hypothesis.

The colorimetric analysis data presented in Figure 1 shows that the KDE had a negative trend in the density curve. This trend was an indication of medium discoloration, which is a marker of TBT biodegradation. DCPIP, a redox dye, changes colour upon reduction, a process linked to the metabolism of microorganisms (Hanson et al. 1993; Bidoia et al. 2010). Therefore, a more negative mean value of the density curve correlates with increased microbial activity, suggesting an association with the tebuthiuron biodegradation process.

At last, the negative values of the LeRR indicate a reduction in TBT concentration, which is a direct result of microbial metabolism. The decrease in TBT concentration corresponds to the microbial activity that was measured by the reduction in DCPIP absorbance. Since TBT was the only available carbon source for the isolated microbial consortia, its degradation was closely linked to the observed changes in absorbance.

The KDE analysis also revealed distinct data distributions, which are consistent with the origins of specific microbial consortia isolates. This means that the distribution of spectrophotometric results, likely related to the medium discoloration, varies among specific microorganisms bioprospected. This variation is attributed to the microbial activities, which are influenced by their genetic composition and the environmental conditions in the sugarcane fields (Moneda et al. 2022).

The values for pC_1_ and pC_2_ clustered near the central region with higher density, while rC_1_ and rC_2_ showed more dispersed values with longer tails. This dispersion in rC was due to the higher organic matter content in the ratoon cane system soil since it provides nutrients to microbial communities, thereby influencing their growth and activity. Therefore, variations in soil organic matter content led to differences in microbial community structure and function, which subsequently impacted the bioremediation process (Bettiol et al. 2023).

The initial statistical modelling approach involved fitting the Gompertz equation to spectrophotometric data for kinetic parameter estimation, referencing Gogoi et al. (2022) and recent studies on tebuthiuron bioremediation (Lima et al. 2022; Nantes et al. 2023). However, the absence of the expected sigmoidal pattern necessitated the application of Welch and Games‐Howell tests for non‐homogeneous variance analysis and mean comparison due to unequal data distribution among conditions.

Welch's Test identified rC_2_ as distinct from the others. This result indicates a higher discoloration rate in the medium with TBT, suggesting that the consortia microorganisms isolated from this sample may be adept at metabolising this herbicide. This could be due to the presence of specific microbial species or functional genes in rC_2_ that are capable of degrading tebuthiuron (Mostafa and Helling 2003).

Furthermore, a Bayesian analysis was conducted to compare the analysed curves. The Bayes factor, calculated using Harold Jeffrey's Default, provided evidence of a difference between the curves. With a 95% confidence interval and a loge [BF] of 2.9, this analysis supports the disparity of the rC_2_ curve, emphasising the potential effectiveness of its microbial community in tebuthiuron bioremediation.

These findings highlighted the importance of microbial bioprospection for pesticide bioremediation. The metabolism of indigenous microorganisms' consortium, a cost‐effective and practical approach, plays a role in this process and interacts with plants in agroecosystems, particularly in the rhizosphere. Therefore, bioprospecting these organisms in agricultural soils is important for enhancing treatment efficiency, as supported by findings from Lima et al. (2022) and Lopes et al. (2022). This study demonstrates the evaluation of the biodegradation potential of tebuthiuron by soil microbial communities under varying management conditions in sugarcane fields.

Finally, there are other methods available for evaluating the biodegradation performance of TBT, such as chromatographic techniques. However, these methods involve high analytical costs, require expensive equipment and demand specialised training for personnel, making them less accessible for routine monitoring, particularly in resource‐limited settings. In contrast, the colorimetric technique proposed in this study offers a more cost‐effective and operationally simple alternative. It does not require advanced instrumentation and allows for rapid assessment of microbial activity. While chromatographic methods provide precise quantification of TBT and its degradation intermediates, this approach focuses on detecting microbial metabolic responses, offering a practical and scalable tool for preliminary screening in biodegradation studies.

### 3.3 Limitations and Strategies for Continued Research

Microorganisms from soils in sugarcane fields with a history of TBT application were proven to form microbial consortia capable of degrading this herbicide. This was confirmed by results, particularly in samples from ratoon cane systems. These microorganisms' metabolic capabilities can be harnessed to develop bioremediation strategies for detoxifying and restoring agroecosystems.

The Gompertz model was initially considered for estimating functional kinetic parameters from the experimental data. However, the colorimetric‐spectrophotometric curves did not follow the expected sigmoidal pattern of microbial activity over TBT presence. This led to the proposal of an alternative analytical methodology that estimates Bayes factors for kernel density curves from the LeRR. The Bayesian‐driven colorimetric protocol addresses the challenges of non‐standard data patterns, providing an alternative for data analysis in bioprospecting studies. This tool significantly distinguishes the physiological action of microorganisms against TBT, potentially contributing to the development of a bio‐based solution for treating this persistent herbicide in large‐scale fields.

Microorganisms from the ratoon cane system (rC), which had higher organic matter due to more intense crop residue deposition on the surface, demonstrated higher efficacy in TBT biodegradation than those from the plant cane system (pC). This indicates that organic matter may influence the concentration and persistence of TBT, potentially leading to extended exposure of soil microbiota and enhancing its ability to degrade this molecule. The research was limited by the lack of detailed characterisation of the microbial communities and soils involved. Understanding how soil properties, especially organic matter content and reactive clays, interact with the herbicide to determine its presence and recalcitrance in sugarcane fields with distinct managements is crucial. These limitations indicate the need for further research to improve the bio‐based solution applicable to bioremediation of agroecosystems.

Future studies should focus on characterising these microbial communities and soil properties in detail, potentially enhancing the efficacy of TBT biodegradation and the applicability of sustainable solutions for bioremediation. Additionally, the next step in the research could include the characterisation of the dominant microorganisms within the consortium to further understand the biodegradation mechanisms involved. This study did not specifically analyse TBT residue concentrations and their degradation intermediates. A kinetic assessment of herbicide degradation would be a valuable addition to future research. Incorporating this aspect into the proposed methodology could provide deeper insights into the biodegradation process and further validate the statistical approach developed in this study.

## 4 Conclusions

The findings support the hypothesis that areas exposed to TBT in Brazil's sugarcane fields may harbour microbial consortia capable of degrading this herbicide. The Bayesian‐based colorimetric assessment revealed a significant reduction in absorbance, indicating the biodegradation potential of indigenous microbial consortia, particularly those in first‐cut ratoon cane fields.

Additionally, the study introduced a Bayesian factor estimation approach for kernel density curves of the logarithmic response ratio, providing an alternative analytical tool for interpreting non‐sigmoidal spectrophotometric data. This method proved effective in transforming raw colorimetric assay data into meaningful insights, facilitating the identification and selection of microbial communities for bio‐based remediation strategies.

To further harness these microbial consortia for bioremediation applications, additional research is required to characterise their composition, metabolic pathways, and environmental interactions. A deeper understanding of these factors will enable the development of tailored and sustainable solutions for mitigating herbicide contamination in agricultural soils.

## Author Contributions

**Letícia Barbosa Jorososki:** conceptualization, methodology, validation, investigation, data curation, writing – original draft. **Bruno Rafael de Almeida Moreira:** conceptualization, methodology, validation, formal analysis, data curation, writing – original draft, writing – review and editing, visualization. **Victor Hugo Cruz:** investigation, writing – original draft. **Yanca Araujo Frias:** investigation, writing – original draft. **Paulo Renato Matos Lopes:** methodology, conceptualization, validation, formal analysis, visualization, writing – review and editing, supervision, resources, project administration, funding acquisition.

## Ethics Statement

The authors have nothing to report.

## Consent

The authors have nothing to report.

## Conflicts of Interest

The authors declare no conflicts of interest.

## Supporting information


**Figure S1.** Microbial growth confirming the bioprospecting process of indigenous soil microbiota with tebuthiuron degradation potential: (A) pC_1_, (B) pC_2_, (C) rC_1_, and (D) rC_2_.

## Data Availability

The data that support the findings of this study are available from the corresponding author upon reasonable request.

## References

[emi470109-bib-0001] Albuquerque, A. F. , J. S. Ribeiro , F. Kummrow , A. J. Nogueira , C. C. Montagner , and G. A. Umbuzeiro . 2016. “Pesticides in Brazilian Freshwaters: A Critical Review.” Environmental Science: Processes & Impacts 18, no. 7: 779–787. 27367607 10.1039/c6em00268d

[emi470109-bib-0002] Almeida, R. L. 2020. “Photosynthetic Capacity in Sugarcane is Limited by Photochemical, Diffusive and Biochemical Factors (Capacidade fotossintética em cana‐de‐açúcar é limitada por fatores fotoquímicos, difusivos e bioquímicos). Master's Thesis (Tropical and Subtropical Agriculture), Campinas, Brazil: IAC, 48.”

[emi470109-bib-0003] Bettiol, W. , C. A. Silva , A. E. P. Cerri , L. Martin‐Neto , and C. A. Andrade . 2023. Understanding Soil Organic Matter in Tropical and Subtropical Environments (Entendendo a matéria orgânica do solo em ambientes tropical e subtropical). Embrapa.

[emi470109-bib-0004] Bidoia, E. D. , R. N. Montagnolli , and P. R. M. Lopes . 2010. “Microbial Biodegradation Potential of Hydrocarbons Evaluated by Colorimetric Technique: A Case Study.” In Current Research, Technology and Education Topics in Applied Microbiology and Microbial Biotechnology, edited by A. Méndez‐Vilas , vol. 2, 1277–1288. Formatex Research Center.

[emi470109-bib-0005] Brazil. Brazilian Institute for the Environment and Renewable Natural Resources . 2020. “Environmental Profile: Tebutiuron. Brasília: IBAMA.”

[emi470109-bib-0006] Correia, N. M. 2018. Behavior of Herbicides in the Environment (Comportamento dos Herbicidas no Ambiente). Vol. 160, 30. Embrapa Hortaliças.

[emi470109-bib-0007] Filizola, H. F. , M. A. F. Gomes , and M. D. Souza . 2006. Manual of Sample Collection Procedures in Agricultural Areas for Environmental Quality Analysis: Soil, Water and Sediments (Manual de Procedimentos de Coleta de Amostras Em áreas agrícolas Para análise da Qualidade Ambiental: Solo, água e Sedimentos). Embrapa Meio Ambiente.

[emi470109-bib-0008] Gogoi, U. N. , P. Saikia , and D. J. Mahanta . 2022. “An Approach to Estimate the Parameters of Hossfeld, Korf and Levakovic‐III Model and Its Application on Tumour Growth.” Advances in Applied Mathematics 22: 311–336.

[emi470109-bib-0009] Hanson, K. G. , J. D. Desai , and A. J. Desai . 1993. “A Rapid and Simple Screening Technique for Potential Crude Oil Degrading Microorganisms.” Biotechnology Techniques 7: 745–748.

[emi470109-bib-0010] Hedges, L. V. , J. Gurevitch , and P. S. Curtis . 1999. “The Meta‐Analysis of Response Ratios in Experimental Ecology.” Ecology 80: 1150–1156.

[emi470109-bib-0011] Kapsi, M. , C. Tsoutsi , A. Paschalidou , and T. Albanis . 2019. “Environmental Monitoring and Risk Assessment of Pesticide Residues in Surface Waters of the Louros River (N.W. Greece).” Science of the Total Environment 650: 2188–2198. 30292989 10.1016/j.scitotenv.2018.09.185

[emi470109-bib-0012] Lima, E. W. , B. P. Brunaldi , Y. A. Frias , B. R. A. Moreira , L. S. Alves , and P. R. M. Lopes . 2022. “A Synergistic Bacterial Pool Decomposes Tebuthiuron in Soil.” Scientific Reports 12, no. 1: 9225. 35655075 10.1038/s41598-022-13147-8 PMC9163133

[emi470109-bib-0013] Lopes, P. R. M. , V. H. Cruz , A. B. Menezes , et al. 2022. “Microbial Bioremediation of Pesticides in Agricultural Soils: An Integrative Review on Natural Attenuation, Bioaugmentation and Biostimulation.” Reviews in Environmental Science and Bio/Technology 21: 851–876.

[emi470109-bib-0014] Mallman, V. , L. W. R. Aragão , S. S. L. Fernandes , T. C. L. Fernandes , R. F. B. Aragão , and R. C. L. Silva . 2019. “The Advantages of Bioremediation in Environmental Quality (As Vantagens da biorremediação Na Qualidade Ambiental).” Ensaios e Ciência 23, no. 1: 12–15.

[emi470109-bib-0015] Mancuso, M. A. C. , E. Negrisoli , and L. Perim . 2011. “Residual Effect of Herbicides in Soil (*Carryover*).” Revista Brasileira de Herbicidas 10, no. 2: 151–164.

[emi470109-bib-0016] Mendes, K. F. , K. C. Mielke , L. H. Barcellos , R. A. de la Cruz , and R. N. Sousa . 2021. “Anaerobic and Aerobic Degradation Studies of Herbicides and Radiorespirometry of Microbial Activity in Soil.” In Radioisotopes in Weed Research, edited by K. F. Mendes , 97–126. CRC Press.

[emi470109-bib-0017] Mendes, K. F. , M. C. F. Wei , I. F. Furtado , et al. 2021. “Spatial Distribution of Sorption and Desorption Process of 14^C^‐Radiolabelled Hexazinone and Tebuthiuron in Tropical Soil.” Chemosphere 264: 128494. 33022507 10.1016/j.chemosphere.2020.128494

[emi470109-bib-0018] Moneda, A. P. C. , L. A. L. Carvalho , L. G. Teheran‐Sierra , M. I. G. Funnicelli , and D. G. Pinheiro . 2022. “Sugarcane Cultivation Practices Modulate Rhizosphere Microbial Community Composition and Structure.” Scientific Reports 12, no. 1: 19174. 36357461 10.1038/s41598-022-23562-6 PMC9649670

[emi470109-bib-0019] Mostafa, F. I. Y. , and C. S. Helling . 2003. “Isolation and 16S DNA Characterization of Soil Microorganisms From Tropical Soils Capable of Utilizing the Herbicides Hexazinone and Tebuthiuron.” Journal of Environmental Science and Health. Part. B 38, no. 6: 783–797. 10.1081/PFC-120025579 14649709

[emi470109-bib-0020] Nantes, L. S. , M. B. Aragão , B. R. A. Moreira , et al. 2023. “Synergism and Antagonism in Environmental Behavior of Tebuthiuron and Thiamethoxam in Soil With Vinasse by Natural Attenuation.” International journal of Environmental Science and Technology 20: 4883–4892.

[emi470109-bib-0021] Pires, F. R. , C. M. Souza , P. R. Cecon , et al. 2005. “Inferences on the Rhizospheric Activity of Species With Potential for Phytoremediation of the Herbicide Tebuthiuron (Inferências Sobre Atividade rizosférica de espécies Com Potencial Para fitorremediação Do Herbicida Tebuthiuron).” Brazilian Journal of Soil Sciences 29, no. 4: 627–634.

[emi470109-bib-0022] Primavesi, A. M. 2017. Some Indicator Plants: How to Recognize Soil Problems (Algumas plantas indicadoras: como reconhecer os problemas de um solo). 1st ed. Expressão Popular.

[emi470109-bib-0031] Qian, Y. , H. Matsumoto , and X. Liu . 2017. “Dissipation, Occurrence and Risk Assessment of a Phenylurea Herbicide Tebuthiuron in Sugarcane and Aquatic Ecosystems in South China.” Environmental Pollution 227: 389–396. 28486182 10.1016/j.envpol.2017.04.082

[emi470109-bib-0023] Rodrigues, B. N. , and F. S. Almeida . 2011. Herbicides Guide (Guia de herbicidas). 6th ed. IAPAR.

[emi470109-bib-0024] Sagarkar, S. , S. Mukherjee , A. Nousiainen , et al. 2013. “Monitoring Bioremediation of Atrazine in Soil Microcosms Using Molecular Tools.” Environmental Pollution 172: 108–115. 23022948 10.1016/j.envpol.2012.07.048

[emi470109-bib-0025] Silva, G. S. , A. F. M. Silva , A. L. Giraldeli , G. A. Ghirardello , R. Victoria Filho , and R. E. B. Toledo . 2018. “Weed Management in the Sugarcane Pre‐Sprouted Seedling System (Manejo de Plantas Daninhas no Sistema de Mudas pré‐Brotadas de Cana‐de‐açúcar).” Brazilian Journal of Herbicides 17, no. 1: 86–94.

[emi470109-bib-0026] Silva Junior, A. C. , C. G. Gonçalves , J. R. G. Queiroz , and D. Martins . 2018. “Evaluation of Leaching Potential of Tebuthiuron Using Bioindicator Plants.” Archives of the Biological Institute 85: e0692015.

[emi470109-bib-0027] Teixeira, M. F. F. , A. A. Silva , M. A. Nascimento , L. S. Vieira , T. P. M. Teixeira , and M. F. Souza . 2018. “Effects of Adding Organic Matter to a Red‐Yellow Latosol in the Sorption and Desorption of Tebuthiuron.” Planta Daninha 36: e018168639.

[emi470109-bib-0029] Wan, R. , Z. Wang , and S. xie . 2014. “Dynamics of Communities of Bacteria and Ammonia Oxidizing Microorganisms in Response to Simazine Attenuation in Agricultural Soil.” Science of the Total Environment 472: 502–508. 24317158 10.1016/j.scitotenv.2013.11.090

[emi470109-bib-0030] Zhang, Q. , L. Zhu , J. Wang , et al. 2014. “Effects of Fomesafen on Soil Enzyme Activity, Microbial Population, and Bacterial Community Composition.” Environmental Monitoring and Assessment 186: 2801–2812. 24362514 10.1007/s10661-013-3581-9

