# Supplementary material for: Bioprospecting Microbial Consortia for Tebuthiuron Degradation in Agricultural Soils: An Alternative Bayesian‐Driven Colorimetric Protocol
Source: Environ Microbiol Rep. 2025 May 26;17(3):e70109. doi: 10.1111/1758-2229.70109 (PMC12105111; doi:10.1111/1758-2229.70109)

**Supplementary Material**

*Description: Photos of Petri dishes with culture medium showing microbial growth that confirms the bioprospecting process of indigenous soil microbiota with tebuthiuron degradation potential*

**Bioprospecting Indigenous Microorganisms for Tebuthiuron Degradation in Agricultural Soils: An Alternative Bayesian-Driven Colorimetric Protocol**

**Figure S1**. Microbial growth confirming the bioprospecting process of indigenous soil microbiota with tebuthiuron degradation potential: (A) pC_1_, (B) pC_2_,(C) rC_1_, and (D) rC_2_.


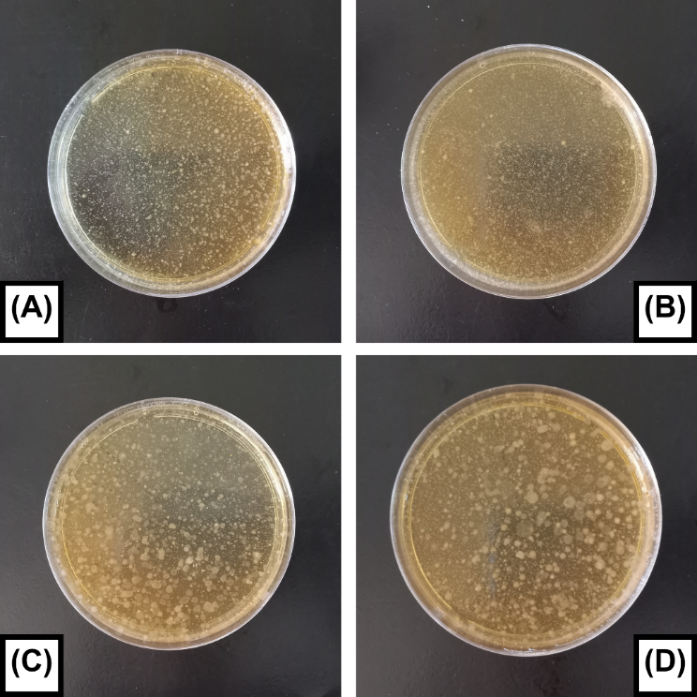

Supplement: Supplementary file 1 — Figure S1. Microbial growth confirming the bioprospecting process of indigenous soil microbiota with tebuthiuron degradation potential: (A) pC1, (B) pC2, (C) rC1, and (D) rC2. [file EMI4-17-e70109-s001.docx]
